# Innovative Damage Assessment of Endodontic Instruments Based on Digital Image Stacking

**DOI:** 10.3390/clinpract15010003

**Published:** 2024-12-26

**Authors:** Raúl Argüello-Sánchez, Ivette Alejandra Calderón-Alday, Antonio Hernández-Morales, Benjamín Gonzalo Rodríguez-Méndez, Diego Medina-Castro, Régulo López-Callejas, Carlo Eduardo Medina-Solís

**Affiliations:** 1 Faculty of Dentistry, Autonomous University of the Mexico State, Av. Paseo Tollocan 13, Colonia Universidad, Toluca de Lerdo C.P. 50130, Estado de México, Mexico; rarguellos@uaemex.mx (R.A.-S.); ahernandezmo@uaemex.mx (A.H.-M.); 2 Stomatology Department of the Mexico State Health Institute, Av. Estado de México S/N, Colonia Rancho Guadalupe, Metepec C.P. 52140, Estado de México, Mexico; alealday3@gmail.com; 3 National Institute for Nuclear Research, Carretera México-Toluca S/N, Ocoyoacac C.P. 52750, Estado de México, Mexico; diego.medina@inin.gob.mx (D.M.-C.); regulo.lopez@inin.gob.mx (R.L.-C.); 4 Academic Area of Dentistry of Health Sciences Institute, Autonomous University of Hidalgo State, Exhacienda de la Concepción S/N Carretera Actopan-Tilcuautla, Tilcuautla C.P. 42160, Hidalgo, Mexico; cemedinas@uaeh.edu.mx

**Keywords:** endodontic files, image stacking, digital images

## Abstract

Background/Objectives: The damage assessment of dental instruments, such as endodontic files, is crucial to ensure patient safety and treatment quality. Conventional scanning electron microscopy (SEM) has been the gold standard for this purpose; however, its limited accessibility and complex sample preparation protocols hinder its routine use in clinical settings. This study proposes a novel system that leverages digital photography and advanced image processing techniques as a viable alternative to SEM. Methods: Our system accurately detects early instrument damage by capitalizing on the high resolution of digital images. Its exceptionally user-friendly interface, portability, and key features make it highly suitable for daily clinical practice. Results: Our findings suggest that the proposed system provides image quality comparable to SEM. Conclusions: Image stacking provides a practical, efficient, and objective method for assessing endodontic instruments’ morphology. By detecting early damage, this system significantly improves the safety and quality of endodontic procedures, especially for reusable NiTi files, instilling confidence and security in its use. It offers a cost-effective and user-friendly alternative to traditional methods such as visual inspection and SEM, making it a comfortable and confident choice for both research and clinical settings.

## 1. Introduction

Excellence in endodontic treatment depends mainly on the quality and maintenance of the instruments used, with endodontic files being crucial pieces in this process. The continuous wear of these instruments compromises the effectiveness of the procedures, which can negatively affect the patient’s treatment [1,2,3].

Over the past two decades, nickel–titanium (NiTi) rotary instruments have revolutionized endodontics and improved root canal cleaning and shaping. Despite their exceptional flexibility compared to stainless steel files, the documented failure rate of NiTi instruments has raised concerns. Several studies have used scanning electron microscopy (SEM) to assess these instruments’ wear and post-use characteristics, revealing that cyclic fatigue fracture is one of the leading causes of failure [4,5]. This coincides with the increased cycles used during endodontic procedures [6]. Currently, several studies have delved deeper into the causes of the fracture of NiTi files in a clinical context, a crucial step towards a deeper understanding of this phenomenon. These findings underscore the urgent need for continued research in this area, highlighting the importance of their work in optimizing instrument performance and significantly improving clinical outcomes [7,8].

Traditionally, endodontic file wear has been assessed using radiography and SEM. While SEM offers exceptional resolution, its implementation in daily clinical practice is limited by the complexity of sample preparation, the cost of specialized equipment, and its availability [9,10,11].

Although SEM can indeed cause some degree of damage to endodontic files, particularly to their sharp edges and surfaces, the extent of this damage can be minimized by adjusting the operating parameters of the microscope. Techniques such as reducing the beam current, increasing the distance between the beam and the workpiece, using a low acceleration voltage, and minimizing exposure time can significantly mitigate this issue. Additionally, employing sample cooling and low-energy detectors can further reduce damage. However, it is essential to acknowledge that any interaction with an electron beam can leave an imprint on the sample, no matter how minimal. Thus, the decision to use SEM for assessing endodontic file wear must be made carefully. It is vital to weigh the benefits of the high resolution offered by this technique against the potential limitations, including sample damage, the necessity for specialized equipment, complex sample preparation, and high costs. These factors may hinder the widespread application of SEM in clinical practice, underscoring the need for alternative methods [12].

The search for a more accessible and accurate analysis procedure has led to exploring new avenues. In this context, high-resolution digital photography and image processing stand out as promising alternatives, offering a bright future for dentistry. They have the potential to capture detailed images of dental instrument surfaces, allowing for the accurate identification of features such as manufacturing defects, fractures, fissures, and abrasive wear, among others [13,14].

Previous studies have employed various techniques to analyze endodontic files, from simple magnifying observation [15] to more sophisticated imaging techniques. Stereomicroscopy [16,17] has been widely used to assess the surface morphology of files, although its resolution is limited. In recent years, micro-computed tomography (micro-CT) [18] has emerged as a powerful tool for obtaining high-resolution three-dimensional images, allowing for the non-destructive assessment of fissures, pores, and changes in the microstructure of files, among others. Micro-CT provides a detailed three-dimensional view of the entire sample. The choice between micro-CT and other techniques, such as SEM or confocal microscopy [19], depends on the specific objectives of each study. While SEM offers an exceptionally high resolution (~1 nm), micro-CT (~1 µm) is a more accessible and versatile alternative for the general assessment of endodontic file integrity.

Image stacking is proposed as a novel and cost-effective technique for evaluating endodontic instruments. This method combines multiple images captured at different focal lengths, allowing for composite images with a greater depth of field and resolution. The potential to obtain high-quality results without needing specialized or expensive equipment is reassuring, offering a hopeful future for dentistry [20,21,22,23].

While image stacking has proven to be a valuable tool in diverse fields such as biology [24], medicine [25], and materials science [26,27], its application in the context of endodontics represents a novel and significant contribution. Unlike other studies that have explored this technique in biological samples or materials, our work focuses specifically on the detailed assessment of endodontic instrument morphology. By taking advantage of digital photography and advanced image processing, our system offers an accessible, efficient, and reliable alternative to traditional techniques such as SEM. This allows for a more frequent and detailed assessment of endodontic instruments, which can improve the quality and safety of endodontic treatments [28,29]. The ability to accurately visualize the surface features of endodontic files and other instruments not only opens new possibilities for research but also holds great promise for enhancing clinical practice, allowing for early damage detection and facilitating more informed decision-making.

This study presents an innovative system that uses high-quality digital image acquisition and image stacking techniques to assess the surface morphology of endodontic instruments in detail. The system’s primary goal is to develop a non-destructive and highly efficient procedure to detect manufacturing defects or use, such as microcracks, fissures, and other issues that could compromise instrument integrity and quality. By providing a more detailed and accurate assessment of endodontic instruments, this system can significantly advance endodontic research and improve the quality of clinical practice.

## 2. Materials and Methods

**Figure 1:**
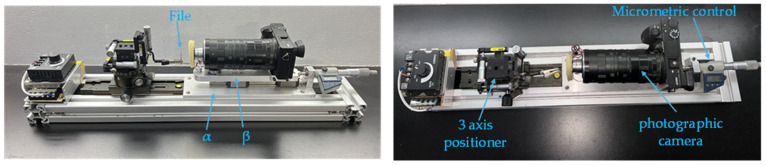
The step-shift-based image stacking system is shown. This system uses a high-resolution Sony A6500 camera (Sony Corp., Tokyo, Japan), a macro lens, and a linear shift mechanism to capture multiple images with a one-micrometer offset between each image.

This section comprehensively describes the multifocal microscopy system, a powerful tool developed to analyze the surface morphology of endodontic files. Figure 1 presents a schematic of the system, which shows the acquisition of images in multiple focal planes. By combining and processing these images, the system overcomes the limitation of the depth of field, allowing for the detailed revelation of surface features such as microcracks, wear patterns, and other defects at the microscopic level. This ability to obtain high-resolution three-dimensional images is essential in detecting possible alterations on the surface of the files, offering a promising potential to significantly improve endodontic treatment outcomes. The specific components of the system, the image acquisition protocol, and the data analysis methods are detailed in the following paragraphs.

A high-precision linear motion platform, meticulously planned and calibrated, is used for image capture. The platform’s base is an aluminum plate (α) with a thickness of 6 mm, length of 260 mm, and width of 75 mm. This plate was carefully calibrated to zero degrees to ensure a flat and uniform surface, which is crucial for the system’s stability and the movements’ precision. A THK linear guide model S.R.S. 7M (T.H.K. Co., Ltd., Tokyo, Japan) was installed with two sliding carriages on this plate. This high-precision linear guide allows for the smooth and controlled movement of the *x*-axis’s secondary plate (β). This precise movement in the *x*-axis is fundamental for capturing sequential images at different focal lengths and essential for image stacking.

The secondary plate (β), with dimensions of 15 mm thickness, 200 mm length, and 40 mm width, was carefully planned and calibrated to zero degrees. This plate, which serves as a mobile platform for the camera, is placed on the sliding carriages of the T.H.K. linear guide. To ensure precise control of the plate (β) movement and to perform micrometric measurements on the *x*-axis, a Mitutoyo 350-712-30 digital micrometer (Mitutoyo Corporation, Kawasaki, Japan) was incorporated. The measuring tip of the micrometer was inserted into the plate (β), allowing for a resolution in the micrometer range.

A high-resolution Sony A6500 (Sony Corp., Tokyo, Japan) camera was installed on the plate (β). Equipped with adjustable extension tubes that allow for the distance between the lens and the camera sensor to be adapted, the camera is a versatile tool for capturing images at different magnification levels. An interchangeable microscope lens was installed at the opposite end of the photosensor, allowing for the magnification to be adjusted according to the needs of the microscopic analysis. This flexible configuration ensures that the system can capture images from a general view of the surface to microscopic details, all with the same high level of quality. The choice of the Sony A6500 camera is based on its high resolution, image quality, and RAW file format capability, which ensures the integrity of the data and is ideal for high-quality image processing.

The complete system was assembled on a robust base constructed from Bosch 4040 aluminum profiles (Bosch Rexroth AG, Lohr am Main, Germany), with dimensions of 650 mm in length and 120 mm in width. This base, made of sturdy and durable aluminum, provides a rigid and stable structure for the system, ensuring stability during image capture. The choice of aluminum for the base was significant as it offers excellent stability and rigidity, which is crucial for the system’s performance. For precise sample positioning, a Parker M400 3-axis positioner (Parker Hannifin Corp., Mayfield Heights, OH, USA) was mounted on a Foto-mate 360 rail (Fotomate International Co., Ltd., Taipei, Taiwan). This system is essential to allow for a precise rotation of the sample and its correct alignment with the camera. The Parker M400 positioner allows for adjustments in all three dimensions (*x*, *y*, *z*), where the *x*-axis is used for horizontal displacement, the *y*-axis adjusts the inclination of the endodontic file to ensure it is perpendicular to the camera capture plane, and the *z*-axis allows for the adjustment of the height of the sample.

However, it is essential to note that image acquisition is performed on a two-dimensional plane despite the system’s three-dimensional capability. This means that once the sample is correctly positioned and aligned, the camera captures the image in only one plane (*x*, *y*), thus ensuring repeatability and accuracy in the sample’s position, which is crucial for clinical analysis. This methodology ensures that the images obtained are consistent and high-quality, facilitating clinical interpretation and diagnosis.

To achieve a uniform and controlled illumination of the sample, an array of light-emitting diodes (LEDs) was used, which are incident on the objective. An external source powers this stereoscopic illumination; the dimmer is the key to its control. The dimmer empowers the user to adjust the illumination intensity according to their needs, ensuring a high level of control. Uniform and controlled illumination is essential to obtain high-quality images with sharp details and without shadows.

The meticulous selection of acquisition parameters is a critical aspect of our system’s operation. To capture the three-dimensional information of a surface, an image stacking technique involves taking a sequence of images at different focal lengths. This process, performed manually, involves panning the camera along the *x*(focus) axis to capture images covering the entire sample depth. The images are then processed using Zerene^®^ Stacker software version 1.04 (Zerene Systems L.L.C., Richland, WA, USA) to create a high-resolution composite image, enhancing the visibility of microscopic features. The quality of the final images is optimized by meticulously selecting acquisition parameters such as resolution, aperture, ISO sensitivity, and file format. This careful selection ensures an extended depth of field and detailed visualization of wear and surface quality, as well as detecting microcracks, fissures, manufacturing defects, and deformation.

The choice of each system component was based on the need for accuracy, stability, and accessibility. The combination of high-precision linear guides, digital micrometers, high-resolution cameras, and specialized image stacking software ensures high-quality images with a precise depth of field. With its stable performance, this system offers an affordable and efficient solution for accurate dental instrument analysis.

To ensure the representativeness of the results, approximately 100 files were selected through a randomization process that included leading brands and others available in the Mexican market, such as FKG, Yahong, Dentsply Sirona, and Coltene. These brands were chosen for their high quality and wide distribution and were specifically selected to reflect a diversity of clinical scenarios. This diversity is based on their prevalence in practice and their compliance with ISO 3630-1 requeriments [30], the international standard for rotary endodontic instruments.

The randomization process was carried out by selecting files from recent production batches, ranging in size from 500 to 1000 units per batch, under controlled conditions. This approach ensured unbiased sampling and minimized the likelihood of bias in instrument selection. The files chosen ranged in lengths of 21, 25, and 31 mm and tapers of 0.04 and 0.06, thus representing standard options commonly used in clinical practice. In addition, we made sure to include root preparation systems with different cross-sectional shapes, allowing for a broad spectrum of instrumentation designs to be reflected and ensuring a comprehensive approach to the study.

**Figure 2:**
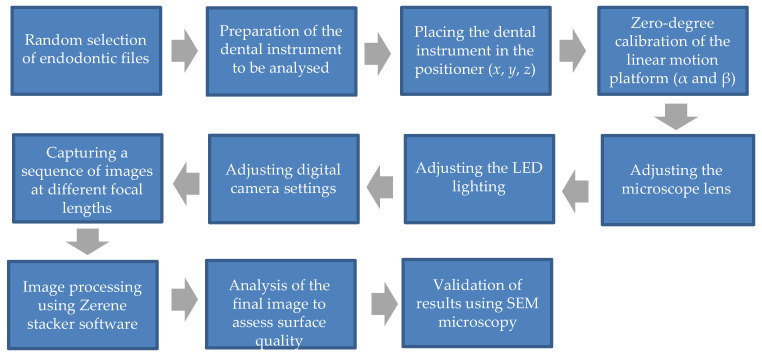
Diagram of the multifocal system to analyze the surface morphology of endodontic files.

To validate the results obtained through image stacking, comparisons were made using a JEOL JSM-5900 LV scanning electron microscope (JEOL Ltd., Tokyo, Japan), operating at 20 kV and 10 pA. The samples were observed at 100× magnification, ideal for detecting microcracks, surface roughness, wear, and other relevant features. This reference technique, crucial in providing a solid comparison point, further confirmed the results’ accuracy and reliability, underscoring the significance of our research. The block diagram of the image stacking procedure is illustrated in Figure 2.

Using 100× magnification is a solid reference point for comparison, offering clear visualization of larger structures while preserving critical details that would be observable at higher magnifications. This magnification choice was established based on a systematic approach to selecting areas of interest (AOIs), including visual inspection and the scientific relevance of specific morphological features. Initially, a preliminary evaluation of the stacked images was performed to identify distinctive morphological features that aligned with the study objectives. AOIs were selected based on criteria such as sample homogeneity to minimize variability, textural features pertinent to the investigation, and areas that exhibited significant differences during preliminary analysis. Additionally, consensus was achieved among multiple observers to validate the selection of AOIs, adding rigor to the process. This standard also facilitates direct comparisons with the image stacking system, which typically operates within a similar resolution range. While higher magnifications may reveal additional detail, they can introduce artifacts and complicate image interpretation, potentially distorting comparisons. Therefore, 100× magnification strikes a balance between resolution and comparability, ensuring the accuracy and reliability of results.

For SEM analysis, the files were installed in aluminum sample holders; AOIs were selected for the images obtained with this system. This selection process was based on the specific areas most indicative of the file’s quality, such as the surface’s roughness. These areas were then compared to ensure a comprehensive evaluation of the files’ characteristics.

To assess the agreement between evaluators in the categorization of qualitative elements, the Kappa index (*κ*) was used. It quantifies the agreement between two or more evaluators, adjusting the agreement observed by chance. The unique ability of the *κ* to go beyond the coincidence expected by chance allows for the degree of agreement between evaluators to be determined, ensuring an objective estimate. This, in turn, significantly improves the quality of the research, demonstrating the value of *κ*.

## 3. Results

The image stacking system developed in this study proved to be an effective tool for the detailed characterization of dental instruments. Multiple high-resolution images of each instrument were captured by the controlled movement of a digital camera equipped with a microscope objective, macro extension tubes, and LED illumination. The results confirm the hypothesis that this technique allows for the visualization of surface characteristics at the micrometric scale, such as microcracks and alterations in surface’s roughness. These findings have practical implications, as they are crucial for evaluating the condition of the instruments and predicting their useful life, thereby contributing to the development and maintenance of dental instruments.

**Figure 3:**
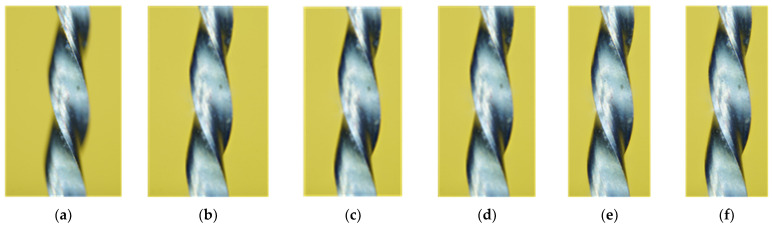
Sequence of photographs of an endodontic file where the focus adjustment was made from 1 to 112 μm to achieve complete coverage of the object, moving the focus point from the closest areas to the furthest areas of the focal plane. The stacking of images obtained with different focal lengths was performed to provide multiple perspectives: (**a**) 1 µm, (**b**) 5 µm, (**c**) 10 µm, (**d**) 20 µm, (**e**) 80 µm, (**f**) 112 µm.

The images obtained using the system shown in Figure 1 allowed for a detailed and precise analysis of the surface characteristics of endodontic files. This method enabled the acquisition of images of the files’ surfaces at different focal lengths, progressing in the resolution with each additional superimposed image. Figure 3 illustrates this process through a sequence of photographs of a new endodontic file, from minimum (Figure 3a) to maximum (Figure 3f) focal length. A comparison of images demonstrates how the superposition of multiple images generates a photograph (Figure 3f) with a much more detailed view of the file’s surface, revealing characteristics that would remain hidden in a single image. This unique capability of the image stacking system is crucial for obtaining an accurate and complete characterization of the endodontic files’ surfaces.

**Figure 4:**
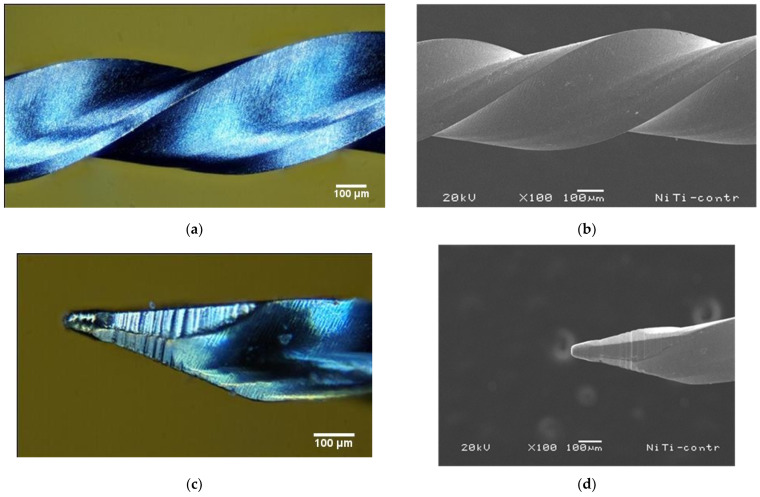
Images of an endodontic file captured with (**a**) the image stacking system at a 200 µm focal length and (**b**) SEM. The images show a similarity in the observed characteristics, highlighting the complementarity of these techniques for analyzing dental instruments at the micrometer scale. The microstructure of the file tip was obtained using two techniques: (**c**) image stacking and (**d**) SEM.

A qualitative comparison was made between images obtained using the image stacking system (Figure 4a,c) and SEM (Figure 4b,d) to thoroughly evaluate the surface characteristics at the micrometric scale. Both techniques allowed us to accurately visualize details such as surface roughness, wear, and the geometry of ridges and grooves. A remarkable similarity was observed in the representation of these morphological features, despite the differences in resolution and operating principles. SEM uses an electron beam to obtain images of the surface, while the image stacking system combines multiple images taken at different depths. The results show that each technique offers specific details about the surface of dental instruments.

The image stacking system’s ability to assess the integrity of dental tools accurately is not just a technical feat but a crucial factor in maintaining treatment quality. The system’s findings, particularly in identifying micrometric-scale morphology such as surface roughness, wear patterns, color changes, microcracks, and fissures, confirm its value in ensuring patient safety and treatment quality.

The image stacking system, comparable to SEM in clinical settings, is advantageous due to its ease of use, accessibility, and speed. Image stacking involves capturing images at different focal planes and combining them to create a single, high-resolution image. While SEM offers a superior spatial resolution and depth of field, enabling detailed 3D visualization, its high cost and complexity limit its accessibility. In contrast, our image stacking system provides a more user-friendly, cost-effective, and versatile alternative (see Figure 4). This system’s versatility allows for the generation of color images that reveal the loss of titanium oxide, a critical factor in the discoloration of sterilized or sodium hypochlorite-immersed files. While reaching a different level of detail than SEM, our system offers a practical and informative solution for applications like endodontic file analysis, ensuring a secure investment.

The image stacking system stands out with its unique ability to capture high-resolution images, going beyond mere visualization. This feature allows for a comprehensive analysis of large sample areas, enabling the identification of wear patterns and microcracks, which might be missed in a more limited analysis. The system’s thoroughness is particularly crucial for evaluating complex instruments, such as endodontic files, where the integrity of each area is vital to the procedure’s success.

The image stacking system’s ability to detect even the minutest changes in endodontic files is a crucial aspect that directly impacts patient safety. This capability also optimizes the management of rotary instruments. By allowing for microscopic analysis, the system enables a precise assessment of the condition of endodontic files (Figure 4), aiding dentists in establishing more efficient maintenance protocols. This knowledge empowers them to identify wear and deformations that can occur in new endodontic files or during use, allowing for their replacement before catastrophic failures occur. The results obtained from this analysis suggest that the durability of endodontic files is not solely determined by the number of times they are used but also by the characteristics of the processed material and the circumstances in which they are used. This emphasis on patient safety should instill confidence in clinical decisions.

**Figure 5:**
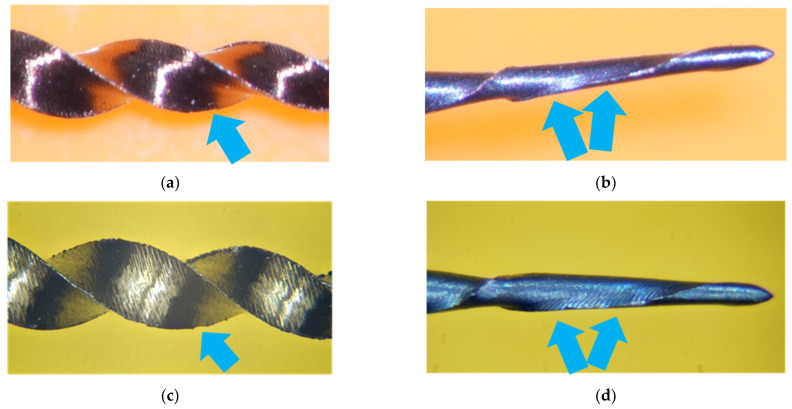
Images (**a**,**b**) were obtained with a dental surgical microscope at 30× magnification, while images (**c**,**d**) were captured using an image stacking system. Figures (**a**,**c**) are associated, as are figures (**b**,**d**). Images (**a**,**c**) show a new endodontic instrument that presents defects on the cutting edge, which are indicated by the arrows. On the other hand, images (**b**,**d**) show an endodontic instrument presenting deformation due to torsion and fracture on the cutting edge, as indicated by the arrows.

A comparison was made between the dental surgical microscope and our image stacking system. Images obtained at the microscope’s maximum magnification of 30× are shown in Figure 5a,b, where relevant structural deformations are evident. However, the detection capacity of the microscope is inherently limited due to its resolution at 30×. In contrast, our image stacking system, as shown in Figure 5c,d, provides a resolution comparable to that obtained at 100× magnification in SEM, enabling a more detailed and precise visualization of the observed structures.

The combination of precision, accessibility, and ease of use makes our image stacking system an invaluable tool for quality control in dentistry. Its ability to detect and characterize damage at the micrometric scale and its versatility to capture high-resolution images make it strategic for optimizing clinical processes. Implementing this technique in clinical settings can pave the way for a future where the quality and safety of dental treatments are even more robust.

**Figure 6:**
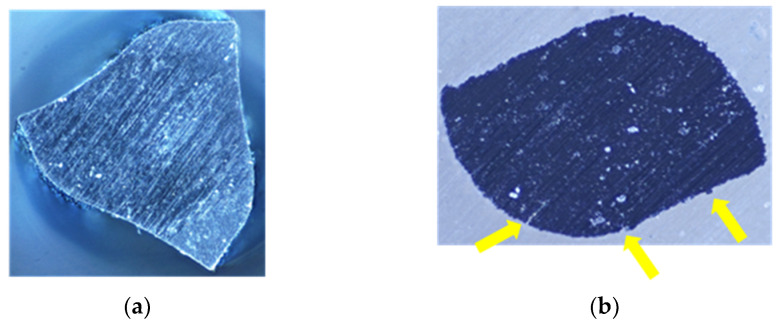
Comparative analysis of the file structure in a cross-section: (**a**) Intact file, showing a homogeneous structure without defects. (**b**) The file exhibits a microcrack, edge wear, and local deformations, with arrows indicating the specific areas of damage.

A cross-sectional microscopic analysis using the image stacking system revealed significant differences in the structural integrity of two endodontic files. While the file in Figure 6a exhibits an intact structure, the file in Figure 6b presents a crack approximately 40 µm wide (indicated by the yellow arrow and measured using ImageJ software 1.54g). This crack compromises the instrument’s strength and rigidity, increasing the fracture risk during use. More importantly, it potentially jeopardizes the procedure’s safety, underlining the gravity of the situation.

**Figure 7:**
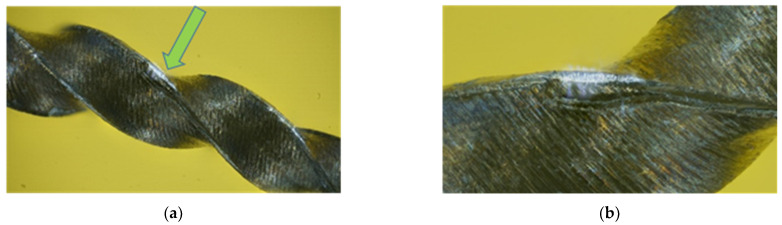
(**a**) General view of a small file segment showing damage to the cutting edges, with the arrow pointing to the affected area. (**b**) Enlargement of the area indicated in (**a**), where a crack can be seen propagating across the edge of the material.

Figure 7 showcases a segment of an endodontic file exhibiting significant damage to its cutting edges. The surface displays pronounced wear and tear, including a bump and a concavity, which compromise the file’s cutting efficiency. A fissure spanning 118 µm further weakens the file’s structure, increasing the risk of instrument breakage. These alterations underscore the urgent need to address the reduced cutting efficiency and increased risk of improper instrumentation, which ultimately impact the quality and safety of endodontic procedures.

By capturing multiple images at different depths of focus and processing them digitally, we ensure the production of high-quality images with an extended depth of field, as shown in Figure 3, Figure 4, Figure 5 and Figure 6. This technique benefits dental files as it allows for a detailed inspection of structures and surface features without needing expensive, specialized equipment.

Table 1 compares both techniques (image stacking vs. SEM). Our image stacking system, with its rapidity, enables image capture within minutes, providing an efficient and time-saving solution. Its portability allows for its use in various settings, further enhancing its practicality. While the image quality may not reach the level of detail provided by SEM, it offers comparable results to 100× magnification, enabling the visualization of micrometric details. However, it is important to note that image stacking is more sensitive to vibrations and changes in illumination.

The results underscore that our image stacking system is an inventive and cost-effective solution for high-resolution image acquisition. Its user-friendliness, adaptability, and competitive cost make it valuable for various applications, from fundamental research to quality control in dental clinics. The system’s ease of use and adaptability ensure a convenient and seamless experience for the user.

The results showed a *κ* value of 0.88, indicating an excellent agreement between both techniques in identifying microcrack and wear features. These findings suggest that the image stacking system is a reliable tool for assessing endodontic instruments’ surface morphology and can be considered a viable alternative to SEM in many clinical applications.

## 4. Discussion

This study led to the development of a novel system to accurately assess the condition of endodontic files using microscopic visualization, a technique of utmost importance in our field. The findings highlight the crucial role of this technique in ensuring the quality and safety of dental treatments. The system provides microscopic visualization that allows for the detection of microscopic damage [31], such as fissures and microcracks, which could compromise the files’ functionality and affect the treatment’s quality. Unlike traditional visual inspections, which may miss these damages, our system offers a more objective and detailed assessment, allowing for more informed decision-making by the clinician. As highlighted in previous studies [32,33], a regular assessment of the conditions of rotary instruments is essential to prevent complications and prolong their lifespan.

This study aimed to develop a non-destructive method based on the image stacking technique to detect microscopic damage to the structure of files, such as microcracks, fissures, and surface wear. This method obtained high-resolution microscopic images that revealed structural details, including those mentioned above. These results have significant practical implications, providing valuable insights into the importance of microscopic evaluation of endodontic files. This knowledge can be used to optimize treatment and improve long-term outcomes [34,35,36,37]. Furthermore, this technique could help compare the performance of different types of files and to assess the impact of different sterilization techniques on the integrity of the instruments.

Our image stacking system offers a practical advantage over SEM with its unique ability to accurately identify texture alterations and microcracks of endodontic files [38,39]. While SEM provides a higher resolution, our system’s global view of the surface makes it a valuable tool for routine instrument assessment in clinical practice. This comparison provides the dental professional with a clear understanding of the practical benefits. Moreover, our system’s accessibility and user-friendliness make it more appealing. These findings underscore the practical benefits of our system as an efficient and accessible alternative to quality control and diagnosis, aligning with current trends in dentistry [24,40,41,42]. Early detection of microscopic damage can play a crucial role in preventing complications and reducing costs associated with returns to the office.

The ability of both systems to detect changes at the microscopic level underlines the importance of assessing the integrity of dental instruments, which translates into a guarantee of patient safety and an improvement in the quality of treatments. However, the complexity and cost of SEM limit its use in daily clinical practice [43,44,45]. In this sense, the stacking system emerges as a viable and accessible alternative, allowing for a fast and accurate assessment of files in the dental office. Its speed ensures an efficient workflow and faster patient care, making it a more practical option than SEM, especially in high-demand clinical environments [41,46,47]. The stacking system can be easily integrated into existing workflows, minimizing the disruption of clinical operations and streamlining workflow for dental professionals.

Our image-stacking system has proven superior to conventional microscopic observation technologies. Our findings indicate that it can achieve magnifications greater than 30× of the dental surgical microscope, providing a higher level of detail and significantly improved resolution, as illustrated in the comparison in Figure 5. This improvement is crucial for visualizing virtually undetectable details with traditional microscopes [48,49]. Although advanced stereomicroscopes can achieve magnification of up to 100×, their high cost (between USD 40,000 and USD 50,000) and limited depth of field represent significant obstacles to their daily use. In contrast, our image stacking system is more affordable. It offers an effective solution for a detailed observation of defects, which may facilitate its adoption in clinical practice, where cost and complexity constraints are common concerns. Our results highlight that this development provides a viable and efficient alternative to existing methods and paves the way for a more optimistic and forward-thinking approach to microscopic observation in dentistry. Most importantly, our system’s ability to improve micro-level defect detection is essential to ensuring the quality and durability of dental instruments, thereby benefiting both professionals and patients by improving patient care.

This study initially focused on a qualitative comparison between the image stacking system and SEM. The results obtained offer a first approximation of the capabilities of both techniques. This exploratory analysis reveals that, at least in the magnification range evaluated, the image stacking system can capture morphological details comparable to those obtained by SEM at a magnification of 100×. However, no previous studies have directly compared the image stacking technique with SEM in the analysis of endodontic files; this exploratory study reveals promising results [50]. However, it is essential to recognize that SEM, due to its operating principle, offers a potentially higher spatial resolution and the ability to obtain information on the chemical composition of the surface [51]. At the same time, the image stacking system presents advantages such as a greater depth of field and the potential for significant advancements in the future. Mainly intriguing is its potential to detect early-stage damage, such as microcracks, fissures, and wear, which can compromise the instrument’s integrity and lead to treatment complications.

The findings of this study have significant clinical implications for the early detection of damage to endodontic instruments, including fractures and wear. This is not just a clinical task, but a strategic one that can significantly impact the financial health of dental practice. This translates into significantly improved patient outcomes and cost savings in clinical workflows for several reasons. Firstly, identifying and addressing these issues in their early stages allows dentists to perform timely interventions that prevent more serious complications. Not only does this avoid invasive and costly treatments, but it also improves the patient’s experience by reducing associated pain and discomfort. A patient who experiences fewer complications and pain has higher satisfaction levels, which is critical to the relationship between the practitioner and the patient. Furthermore, this proactive approach optimizes the use of clinical time. By resolving minor issues early, dentists can see more patients without compromising the quality of care. This is especially relevant in a clinical setting where efficiency is key to the practice’s success. From an economic standpoint, early damage detection prevents progression to conditions requiring more complex and costly procedures. This results in significant savings for both patients and practices, as the cost of a preventative treatment is often lower than that of an advanced complication. In short, early detection not only improves clinical outcomes and patient satisfaction but also results in significant financial savings, supporting the viability and effectiveness of this approach in dental practices.

Despite the significant advantages of the image stacking system regarding accessibility and cost-effectiveness, it is essential to consider some limitations that may influence its clinical implementation, especially regarding endodontic files. First, the system may be sensitive to vibrations, which could affect the quality of the images obtained and, consequently, the accuracy of the diagnosis. Vibrations during the capture process may introduce irregularities into the images, making it difficult to interpret the conditions of endodontic files accurately. Furthermore, consistency in lighting is another critical factor to consider. Variations in ambient lighting during image capture may result in discrepancies in image quality, affecting the practitioner’s ability to assess endodontic files properly.

The choice between image stacking and SEM for evaluating endodontic files is a matter of balancing the advantages and limitations of each technique. The image stacking system, with its accessibility and minimal training requirements, is a practical choice for routine evaluation [52]. However, its performance can be significantly influenced by factors such as vibration, non-uniform illumination, and the challenge of achieving perfect focus across the entire sample, which can limit the resolution and quality of the images obtained [53]. In contrast, SEM, with its exceptional resolution and considerable depth of field [54], may not be suitable for detecting microscopic defects smaller than tens of nanometers. The stacking system can also introduce software issues [55], limit 3D visualization [56], be sensitive to contamination [57], and struggle with opaque materials [58]. Despite these limitations, using the image stacking system improves the detection of microcracks and surface wear on endodontic files and has significant implications for clinical practice. Firstly, accurately visualizing the condition of files allows clinicians to make more informed decisions about the need for instrument replacement or adjustment before complications occur. This helps prevent problems during treatment and contributes to patient safety. On the other hand, using the image stacking system can optimize procedure time by reducing the need for corrective interventions for file fractures. This makes the process more efficient and results in a more satisfying experience for the patient by minimizing treatment duration and complexity.

Compared to the findings obtained by image stacking, which showed comparable results to those of a SEM at 100× magnification, it is essential to consider other non-destructive evaluation methods applied in dental instrument research. For example, X-ray computed tomography (CT) effectively assesses the structural integrity of endodontic files without causing damage to them. This method allows for a three-dimensional visualization of internal structures, facilitating the analysis of possible file failures or wear [59]. Furthermore, techniques such as nuclear magnetic resonance (NMR) [60], micro-CT [61], and cone beam computed tomography (CBCT) [62] have been used to assess the quality of instruments without compromising their integrity, offering a valuable alternative to invasive methods. These approaches minimize the risk of damage to endodontic files and provide detailed information on their functional status, which is crucial to optimize their use in clinical treatments.

Implementing the damage assessment system on dental instruments, especially endodontic files, has significant practical applications in the dental clinic. A key area is routine quality control, where patient safety and treatment effectiveness are essential. The proposed system allows for damage to be detected on new and reusable instruments, such as NiTi files, facilitating a rapid and objective assessment before each procedure. This optimizes treatment quality and minimizes the risk of complications by ensuring the use of instruments in optimal conditions. Furthermore, the system is valuable in the training of new endodontic professionals. By using high-resolution images and damage analysis, students can identify alterations in instrument morphology, developing a critical eye essential for their future clinical practice. Integrating theory and practice in an enriching learning environment significantly enhances training. The system’s portability and ease of use make it suitable for mobile clinics or community dental care, where access to more complex technologies may be limited, thus offering an accessible and effective alternative to SEM. Based on the above, it can be stated that the proposed system provides an innovative solution that responds to the current needs of the dental community and establishes a new standard in evaluating the quality and safety of endodontic instruments.

## 5. Conclusions

The results of this study underscore the practicality and efficiency of the image stacking system as a tool for assessing the surface morphology of endodontic instruments. This system, which provides an objective and detailed assessment of the morphology of endodontic files, uses high-resolution images to detect early microscopic damage, such as microcracks, fissures, and wear patterns. The early detection of such damage is a crucial feature of the system, significantly improving the quality of endodontic procedures and contributing to patient safety, especially by early identification of defects in reusable NiTi files. The image stacking system, with its more objective and detailed assessment compared to traditional methods, has the potential to significantly improve the quality and safety of endodontic procedures. While scanning electron microscopy (SEM) remains the gold standard for high-resolution imaging, the image stacking system provides a more accessible and cost-effective alternative, particularly for routine clinical applications. Its easy-to-use and portable nature make it a promising tool for research and clinical practice. It is crucial to consider the limitations of the system, as addressing these is essential to optimizing its use and ensuring its effectiveness in clinical practice. For example, sensitivity to vibrations can affect the quality of the images obtained, compromising the accuracy of diagnosis and treatment. Furthermore, inconsistencies in illumination can cause variations in the visualization of endodontic files, which could lead to misinterpretations. In conclusion, the image stacking system represents an innovation in evaluating endodontic instruments, and its correct implementation and adaptation to clinical conditions are fundamental to maximizing its benefits.

## Figures and Tables

**Table 1:** Comparison of the main characteristics of the image stacking system and SEM.

Feature	Image Stacking System	Scanning Electron Microscope
Resolution	Of the order of tenths of nm, through which an observation of the microcracks of the files is achieved	From 1 nm, allowing for the observation of atomic-scale structures
Image acquisition time	5 min	20 min
Sample preparation	Remarkably simple, requiring basic cleaning and proper assembly; the mount is one-piece and allows for rotation	Complex: dehydration, metallization, and requires vacuum equipment
Cost per analysis	<1 dollar	USD 25 per image
Portability	Portable, 25 cm × 80 cm × 20 cm	Stationary laboratory equipment with an area of more than 5 m^2^
View field	Large, ideal for viewing the entire surface	Limited, ideal for high-resolution details
Ease of use	Easy-to-operate, intuitive interface	Requires specialized training
Applications	Surface evaluation, detection of microcracks	High-resolution analysis, chemical composition
Equipment cost	Around USD 3000	Thousands to millions of dollars

## Data Availability

The original contributions presented in this study are included in the article. Further inquiries can be directed to the corresponding author.
